# The Alabama VIP older driver study rationale and design: examining the relationship between vision impairment and driving using naturalistic driving techniques

**DOI:** 10.1186/s12886-018-0686-5

**Published:** 2018-02-07

**Authors:** Cynthia Owsley, Gerald McGwin, Jonathan F. Antin, Joanne M. Wood, Jennifer Elgin

**Affiliations:** 1 Department of Ophthalmology, School of Medicine, University of Alabama at Birmingham, Birmingham, AL 35294-0009 USA; 2 Department of Epidemiology, School of Public Health, University of Alabama at Birmingham, Birmingham, AL 35294-0022 USA; 3 Center for Vulnerable Road User Safety, Virginia Tech Transportation Institute, 3500 Transportation Research Plaza, Blacksburg, VA 24060 USA; 4 School of Optometry and Vision Science, Queensland University of Technology, Park Road, Kelvin Grove, Brisbane, VIC Australia

**Keywords:** Vision, Vision impairment, Driving, Aging, Motor vehicle collision, Naturalistic driving

## Abstract

**Background:**

Older drivers aged ≥70 years old have among the highest rates of motor vehicle collisions (MVC) compared to other age groups. Driving is a highly visual task, and older adults have a high prevalence of vision impairment compared to other ages. Most studies addressing visual risk factors for MVCs by older drivers utilize vehicle accident reports as the primary outcome, an approach with several methodological limitations. Naturalistic driving research methods overcome these challenges and involve installing a high-tech, unobtrusive data acquisition system (DAS) in an older driver’s own vehicle. The DAS continuously records multi-channel video of driver and roadway, sensor-based kinematics, GPS location, and presence of nearby objects in front of the vehicle, providing an objective measure of driving exposure. In this naturalistic driving study, the purpose is to examine the relationship between vision and crashes and near-crashes, lane-keeping, turning at intersections, driving performance during secondary tasks demands, and the role of front-seat passengers. An additional aim is to compare results of the on-road driving evaluation by a certified driving rehabilitation specialist to objective indicators of driving performance derived from the naturalistic data.

**Methods:**

Drivers ≥70 years old are recruited from ophthalmology clinics and a previous population-based study of older drivers, with the goal of recruiting persons with wide ranging visual function. Target samples size is 195 drivers. At a baseline visit, the DAS is installed in the participant’s vehicle and a battery of health and functional assessments are administered to the driver including visual-sensory and visual-cognitive tests. The DAS remains installed in the vehicle for six months while the participant goes about his/her normal driving with no imposed study restrictions. After six months, the driver returns for DAS de-installation, repeat vision testing, and an on-road driving evaluation by a certified driving rehabilitation specialist (CDRS). The data streams recorded by the DAS are uploaded to the data coordinating center for analysis.

**Discussion:**

The Alabama VIP Older Driver Study is the first naturalistic older driver study specifically focused on the enrollment of drivers with vision impairment in order to study the relationship between visual dysfunction and driver safety and performance.

## Background

Driving is a highly visual task [1, 2]. Yet vision impairment is common in older adults [3, 4], and thus an important question is how vision impairment impacts older driver safety. Older drivers aged ≥70 years old have among the highest rates of motor vehicle collisions (MVC) compared to drivers in other age groups [5]. Research over the past 2–3 decades indicates that some types of vision impairment are associated with elevated MVC risk in older drivers including slowed visual processing speed [6–8], visual field defects [8–10], and contrast sensitivity impairment [11]. The majority of studies addressing risk factors for crash involvement, including population-based studies, have utilized accident reports as the primary outcome, which are submitted by police who typically do not directly witness the crash. While accident reports document that a crash occurred and provide a wealth of information on the circumstances (e.g. place, weather, vehicles involved, driver’s age), they cannot always be used to provide an accurate description of what actually happened or the causes of the crash. Additionally, accident reports do not provide information on the occurrence of crash events where police do not attend the scene, collisions occurring on private property (e.g., parking lots), and near-misses; thus outcome events are likely incomplete and biased [2]. Previous studies using accident reports to identify risk factors for collision involvement do not objectively measured driving exposure (miles driven), but rather have relied on the driver’s self-report of driving exposure. Such studies cannot address mechanistic questions about how impaired visual function directly impacts driving performance. They provide little to no information about how visual function is related to driver behaviors and vehicle control, such as lane control and turning, or the impact of secondary tasks on driver behavior and vehicle control. All these issues undermine the goal of achieving a comprehensive understanding of visual mechanisms underlying driver performance and safety.

Instead of relying on accident reports as the outcome of interest in studying the relationship between vision impairment and driver safety and performance, in the present study we use naturalistic driving research techniques [12, 13]. This approach involves installing a high-tech yet unobtrusive data acquisition system (DAS) in a participant’s own vehicle. The DAS continuously records multi-channel video of the driver and roadway environment, sensor-based kinematics data, GPS location, and presence of nearby objects near the front of the vehicle. The DAS’s unobtrusive design is facilitated by advances and miniaturization of computer, sensor, data storage, communications, and video technology. It is designed to automatically and continuously collect data whenever the instrumented vehicle is driven by the research participant (i.e., from key on to key off), and remains installed in the vehicle for a lengthy period of time (i.e., months or even years). The advantages of naturalistic driving techniques are striking in contrast to other driving research methodologies. Naturalistic methods avoid the short snapshot (e.g., 1 h) of a standardized course of on-road driving typical of most on-road studies, where drivers know they are being evaluated by study personnel and thus are likely to be on their “best behavior” [2]. On the other hand, such in-person evaluation may also result in poorer performance if the perceived pressure of the study participation causes stress. Also, the route driven during the on-road evaluation may not be representative of the typical driving trips (e.g., traffic density, types of roadways) made by study participants in their everyday life. In naturalistic research, the driver chooses all driving routes in the course of everyday life. On-road evaluation includes explicit instructions by the CDRS on when and where to make turns (e.g., “at the next traffic light, turn left”). Naturalistic driving methods allow for the study of not only crash events but near-crash events, which are similar in terms of driver behavior and vehicle kinematics to actual crashes, yet occur at a rate 2–10 times higher than crash events [14, 15], thus creating a larger number of outcome events to analyze in risk factor modeling.

Naturalistic driving methods have been successfully employed in the study of driver safety and performance for over 10 years, a body of work that establishes their feasibility as a measurement approach. The literature on naturalistic driving research specifically focused on older drivers is, however, small and is summarized here. Older adults who experience a decline in contrast sensitivity over 12 months are more likely to be involved in rapid deceleration events while driving [16]. Visual function in older drivers was found to be unrelated to involvement in lane changing errors [17] but narrowing of the visual attentional field was associated with a higher risk of failing to stop at red lights [18]. Older drivers who restrict their night driving tended to be those with worse visual fields and contrast sensitivity [19]. With respect to head movement while driving through intersections, older drivers had a greater degree of lateral head rotation than middle-aged drivers [20], with the authors suggesting that it may be a compensatory mechanism for older adults’ reduced visual attention skills. Only a few studies thus far have used naturalistic driving data to study visual risk factors for crash and near-crash involvement by older drivers. Older drivers with worse contrast sensitivity had a higher rate of crash and near-crash events [21], however this finding was based on only 20 drivers. Using the Strategic Highway Research Program 2 (SHRP 2) data [13], two studies using different analytic approaches both found that impaired contrast sensitivity and peripheral vision were related to elevated rates of collision involvement [15, 22]. Also using SHRP 2 data, Guo et al. [23] found that secondary-task-induced distractions posed a greater safety threat for older drivers than for middle-aged drivers, however older drivers were less likely to be engaged in secondary tasks while driving. Studies based on the SHRP 2 data included participants with normal or near-normal visual sensory and visual-cognitive skills, thus making it difficult to evaluate associations between visual dysfunction and driver safety and performance.

Here we describe the Alabama VIP Older Driver Study, a naturalistic driving study designed to examine associations between vision impairment in adults ≥70 years old and crash and near-crash involvement as well as other driver behaviors. In order to overcome a major limitation of earlier studies as described above (i.e., most had normal or near-normal vision), our enrollment process targets older adults with a range of visual capabilities with respect to contrast sensitivity and visual processing speed. These aspects of vision were selected as enrollment criteria because they are two of the strongest visual risk factors for collision involvement and driving problems in older adults [6–8, 11, 16, 24, 25]. Our recruitment strategy also targets older adults who are patients from an ophthalmology clinic since they are more likely to have chronic eye conditions that cause visual impairment. The study has three aims:

- Aim 1

To examine the relationships between vision and naturalistic driving performance in older drivers ≥70 years old. Analyses will focus on the relationship between vision and safety critical events (crashes, near-crashes), lane-keeping, turning at intersections, driving performance under secondary task demands, and when a “co-pilot” (passenger in the front seat) is present. Visual function measurements will include assessments of contrast sensitivity, visual processing speed, visual acuity, visual field sensitivity, and visuo-spatial processing. These aspects of vision were selected because they have been widely related to older driver safety and performance [6–11, 16, 24–29]. There are several hypotheses relevant to this aim. Older drivers with contrast sensitivity loss, slowed visual processing speed, and/or visual field impairment will be more likely to exhibit critical safety events, lane keeping deviations, and intersection turning errors, as compared to those without these impairments. Older drivers with these vision impairments will be more likely to exhibit these problems under secondary task demands than drivers without these vision impairments. Older drivers with vision impairment who have a co-pilot will be less likely to exhibit these problems than drivers with vision impairment who do not have a co-pilot.

- Aim 2

To examine these relationships in light of potential effect modifiers, specifically, driver characteristics (e.g., other visual problems, cognitive status, medical conditions, recent history of MVC, physical function, medications); environmental factors (e.g., roadway type, weather, time of day); and vehicle factors (e.g., type of vehicle, tire condition). Our primary hypothesis here is that drivers with both visual impairment and cognitive impairment will be more likely to exhibit critical safety events, lane keeping deviations, and intersection turning errors, as compared to those without vision impairment only but not cognitive impairment.

- Aim 3

To examine the relationships between driving performance as measured by naturalistic driving methods and driving performance ratings provided by a CDRS [30] on a standardized driving route (the clinical gold standard). Our primary hypothesis is that worse CDRS ratings of driving fitness will be associated with more lane keeping deviations, intersection turning errors, and rapid decleration/acceleration events.

## Methods/design

### Overview

**Fig. 1:**
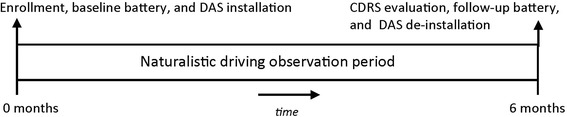
Study baseline and follow-up period: Following screening to establish eligibility, there is a baseline enrollment visit consisting of administration of the health and functioning battery and installation of the data acquisition system (DAS) in the participant’s vehicle. The participant then returns home with his/her vehicle and goes about driving as they normally would during the course of everyday life for a six-month period. Six months following installation the participant returns for the follow-up visit which consists of repeat vision screening, an on-road driving evaluation by a certified driving rehabilitation specialist (CDRS), and de-installation of the DAS

This is a prospective cohort study on older drivers ages ≥70 years old. The study was approved by the Institutional Review Boards of the University of Alabama at Birmingham (UAB) and Virginia Tech Transportation Institute (VTTI) and follows the tenants of the Declaration of Helsinki. The study design from the standpoint of participant flow through the protocol is displayed in Fig. 1. Following written informed consent, participants undergo a testing battery to assess vision, cognitive status, general health, depression, physical functioning, and medications at the UAB Department of Ophthalmology’s Clinical Research Unit. Within one week of enrollment, the DAS is installed in the participant’s vehicle by specially-trained staff in a garage outfitted with custom tools and fixtures to support the installation, alignment, and calibration of each DAS. Following installation, participants then go about driving in their daily lives for six months during which period the DAS continuously records the data streams as previously described. During the six-months period the DAS is remotely and unobtrusively monitored by an integrated team of VTTI and UAB technicians to ensure proper operation. If problems with DAS functioning are detected, the team performs a triage to determine how to address it, thus ensuring the highest quality data possible. At the end of the six months the participant returns to the garage facility at which time the DAS is de-installed. While the DAS is being de-installed, the participant undergoes an on-road evaluation by the CDRS. We also repeat contrast sensitivity testing and visual processing speed testing to assess whether these aspects of vision have changed during the six-month period. All protocol details are below in the section “Study Protocol”.

### Source population

This study focuses on older drivers ≥70 years old at enrollment. We have selected this age group for study for 2 reasons: (1) Motor vehicle collision rates are at their highest for this age group of drivers (with the exception of 16–25 year-old drivers) [5]; and (2) vision impairment rates are at their highest in adults aged ≥70 compared to all other age groups [3, 4]. We are using two recruitment sources for this study: (1) Persons who participated in a previously conducted population-based study on older drivers in our region (*N* = 2000) [31]; (2) Patients seen in the Callahan Eye Hospital Clinics with ocular conditions that cause vision impairment. Potential participants are assessed for inclusion and exclusion criteria at an initial screening visit. Inclusion criteria for enrollees are: (1) age ≥ 70 years old at enrollment; (2) must hold and provide proof of current State of Alabama driver’s license; (3) is a current driver defined as driving at least 4 days per week by self-report; (4) speaks English; (5) currently owns a motor vehicle; (6) is willing to provide permission to install a DAS in the vehicle for six months. Exclusion criteria are: (1) has a planned period that prevents driving for more than 2 consecutive weeks (e.g., planned hospitalization, vacation) within the six-months study period; (2) has a vehicle that is incompatible with the DAS installation per a list of incompatible makes/models and an inspection by the trained installers.

### Study protocol

A screening visit is scheduled whose purpose is two-fold: (1) to provide general information about the study and what participation involves, and (2) to determine the participant’s contrast sensitivity and visual processing speed scores. Our eventual sample of older drivers will be.

stratified with respect to the presence and extent of contrast sensitivity impairment and slowed visual process speed, so that in the final sample, there will be a range of these abilities represented. Table 1 is the stratification grid. Our target sample size is *N* = 195; our goal is for the sample to be approximately evenly distributed throughout the stratification cells (21–22 participants in each cell). Rationale for sample size and statistical power are discussed below in the Statistical Analysis section.

**Table 1:** Stratification for enrollment; target *N* = 195^a^

	Categories for visual processing speed scores (msec)^b^		
Categories for contrast sensitivity scores (log sensitivity)^c^	< 150	150–350	> 350
≥ 1.5			
≥ 1.25 to < 1.5			
< 1.25			

^a^21–22 participants in each cell

^b^Visual processing speed measured by UFOV subtest 2

^c^Contrast sensitivity measured by the Pelli-Robson chart

If the potential participant and his/her vehicle meet the study’s eligibility criteria at the screening visit, a baseline enrollment visit is scheduled within 1–2 weeks. There are two components to the baseline line visit – administering the battery of health and functioning interviews/testing including vision screening at the Clinical Research Unit in the Callahan Eye Hospital and installation of the DAS in the participant’s vehicle at the garage. The health and function battery consists of the following components. These assessments were specifically selected since they have previously linked to older driver safety and performance [1].

### Vision screening

Distance visual acuity is measured binocularly using the Electronic Visual Acuity (EVA) tester and its standard protocol [32] under the participant’s habitual viewing conditions when driving (i.e., with the spectacle correction they wear habitually for driving, if any). Results for analytic purposes are expressed as logMAR. Visual field sensitivity is assessed for each eye separately using a custom test for the Humphrey Field Analyzer (HFA) Model II-I, developed for a previous study [9, 33]. Light sensitivity will be measured using the HFA’s full-threshold procedure and white stimulus-size III targets presented at 20 visual field locations selected to be those that fall within the visual field area relevant when a driver gazes down the road through a vehicle’s windshield or to the vehicle’s dashboard [34]. Since driving is performed using both eyes together, following testing the monocular fields from each participant are combined to form a binocular field consisting of 21 points spanning 60° to the right and left, and 15° to the superior field and 30° to the inferior field. The sensitivity at each test location is defined by the more sensitive point (higher value in decibel units) of the two eyes. [35] Contrast sensitivity is measured binocularly with the Pelli-Robson chart using its standard protocol [36] and scored by the letter-by-letter method [37]. Scores are expressed in terms of log contrast sensitivity. Visual processing speed is measured binocularly using UFOV® subtest 2 [38, 39]. This computerized test provides an estimate of a person’s visual processing speed while identifying a target in central vision and simultaneously localizing a peripheral target at 10° eccentricity in any of 8 radial directions. We also assess visual processing speed using the Trail making Test Part B test [40], a paper-and-pencil test; Trails B performance also relies on executive function and working memory skills. It is a “connect the dots task” with two sets of dots, one labeled from 1 to 25 and the other labeled A to Z. The participant connects the dots by alternating between numbers and letters (i.e., 1, A, 2, B, 3, C, etc.). Performance is expressed in terms of the time to complete the test. The visual closure subtest of the Motor-free Visual Perception Test (MVPT) [27] is used to assess visual-spatial processing ability; the test assesses the ability to match pictures of objects with incompletely drawn pictures of the same objects.

### Other health and functioning assessments

General cognitive status is evaluated using the Mini-Mental Status Screening Exam (MMSE) [41], a valid and reliable screener for cognitive impairment. Depression is assessed using the Center for Epidemiological Studies – Depression (CES-D) scale [42], a validated and reliable screener for depressive symptoms in the elderly. We will administer two physical function tests -- bilateral hand grip strength as measured by the Jamal dynamometer [43], and the Get Up and Go test [44], a test of balance and functional mobility designed for older adults. Participants are asked whether they have fallen in the previous 12 months, and if yes, how many times. Medical conditions are identified by a general health interview used extensively in our previous work [45, 46] that asks about the presence or absence of problems in 17 areas (e.g., heart disease, cancer, diabetes). Participants are asked to bring any current medication containers to their visit, and we inventory all current prescription and over-the-counter medications and dosage regimens;

### DAS installation

While the participant is undergoing the testing protocol for the baseline visit, the DAS is installed in the participant’s vehicle by trained personnel at the study’s garage. Participants are informed that the DAS does not impact the operation of their vehicle and that their experience in driving and controlling the vehicle will be identical to that when the DAS is not present. Upon completion of installation, participants are instructed to drive during the course of everyday life just as they would normally drive. The DAS remains installed in the participant’s vehicle for six months, at the end of which they are scheduled for a DAS de-installation and follow-up visit. In the event participants need to reach the study coordinator with questions during the six-month follow-up interval, they are provided with the coordinator’s telephone number.

### Data acquisition system (DAS)

The DAS, its installation and its operation, is completely compatible with the vehicle. It derives its power from the vehicle. As mentioned earlier, the presence of the DAS in the vehicle does not impact the vehicle’s operation, nor does it impact the way that the driver “experiences” or controls the vehicle. The DAS is largely unobtrusive for the driver and passengers in the vehicle. The video camera unit mounted behind the rearview mirror is visible to the driver, but it is mostly obscured by the rear-view mirror. It is important to note that there is ample evidence from the hours and hours of video recorded on many drivers in the 100-Car study and the SHRP 2 study [12, 13] that drivers appear to “forget” the camera is there. Past participants whose vehicles were outfitted with the same or similar DAS units displayed a host of behaviors that one would not typically display in front of other people or if one was concerned with being video recorded. The system automatically starts up when the vehicle is started (i.e., from key on) and shuts down when the vehicle is turned off (i.e., key off). The participant does not have to do anything to start the DAS’s recording function, nor does the participant have to do anything to calibrate or otherwise interact with the device. The DAS is reliable in many types of inclement weather and also in very hot or cold weather. An important feature is that the “health” and functioning of the DAS is checked remotely and automatically by VTTI through cell phone networking technology, and if there is a malfunction, VTTI technicians detect it, and can communicate it to UAB staff so local technicians can implement a repair process. The DAS’s sensors and recording devices used in this study are the same as those used in the SHRP 2 study [13] and include the following: 5-channel video recording (driver’s face, over the shoulder to dash views, front view, passenger view, rear view), accelerometers, GPS, forward radar, ambient illumination sensor, infrared illumination of the face at night or in dim conditions, turn signal recording, and the following vehicle network data: accelerator position, brake actuation, and speed).

### Six-month follow-up visit

The follow-up visit has three components. One component is to repeat vision screening. The purpose of this screening is to determine if during the ensuing six months, there have been significant visual changes since enrollment. Rather than repeat the entire lengthy vision battery, we have selected two aspects of vision for re-screening at follow-up that the literature suggests are most closely tied to older driver safety and performance, namely contrast sensitivity and visual processing speed using UFOV® subtest 2 [6–8, 11, 24].

The second component consists of an on-road driving assessment. A CDRS who is also a licensed occupational therapist with certificate training in visual impairment, completes an on-road driving assessment of the participant in the UAB driving assessment clinic vehicle. This type of evaluation is widely accepted as the clinical gold standard for assessing driving fitness in older adults who may be medically or functionally compromised. The assessment takes place on a standard route between 9 am and 3 pm on weekdays for all drivers and lasts about 45–60 min depending on traffic volume. The route is about 15 miles and goes through a variety of roadway environments (commercial areas, residential neighborhoods). The clinic’s vehicle is used since it is equipped with a side-brake in the front passenger-seat so that the CDRS (who is seated there) can control the vehicle, if necessary, in the interest of preserving safety; using a clinic vehicle is also considered to be standard clinical practice. The CDRS rates the participant’s driving using a rating scale whose basic structure and components are commonly used by many CDRS’s [47–50], where five skill components are evaluated, each on a 5-point scale: interaction–communication with other road users and pedestrians, driving style (margin of anticipation), vehicle control skills (smoothness), adjustment to traffic speed conditions, reaction to unexpected events, and unusually bad driving maneuvers (e.g., stopping in a lane on the interstate, turning the wrong way on a one-way street). This rating scale is described in detail elsewhere [47]. The CDRS uses the same scale to generate a rating of overall driving performance and makes a clinical judgment as to whether the driver has the potential for safe driving (yes with no restrictions, yes with some restrictions, no). The clinic vehicle is installed with the same DAS as the participants’ vehicles, so that the same objective recordings of vehicle sensors and driver behavior can be made. Having both the CDRS’s ratings and the objective DAS recordings will allow us to examine their relationship.

The third component of the follow-up visit involves de-installation of the DAS from participant’s vehicle at the garage. This occurs while the participant is completing the vision screening and on-road assessment.

### Driver safety and performance variables

We will focus on five aspects of driver safety and performance when evaluating the DAS data as described below.

#### Safety critical events

The most direct measure of driver safety is assessment of events that threaten the safety of the driver and other road users. We will study two types. (a) Crashes, which are defined as any contact that the subject vehicle has with an object, either moving or fixed, at any speed in which kinetic energy is measurably transferred or dissipated, and (b) Near-crashes, which include any circumstance that requires a rapid, evasive maneuver by the subject vehicle, or any other vehicle, pedestrian, cyclist, or animal to avoid a crash; a rapid, evasive maneuver is defined as a steering, braking, accelerating, or any combination of control inputs that approaches the limits of the vehicle’s capabilities.

##### Data reduction

Crash-related events are identified via a variety of means using the DAS time-series data described in detail elsewhere [51]. VTTI research staff first perform a driver identification task to determine which trip files were produced by the consented participant. Trip files produced by others are removed [52]. Then, trained VTTI analysts review video data when vehicle physical sensors detected (1) large changes in speed or position of the car with respect to the road, (2) the participant pushed the critical incident button to flag an event, or (3) the analysts detected a safety critical event [51]. A short window of video surrounding the possible event is extracted and reviewed by trained analysts at VTTI to verify and classify as a crash or near-crash event [53]. The VTTI analysts coding crash and near-crash events are unaware of the participant’s status on any variables collected at the enrollment visit. Data reduction training takes approximately two weeks. During this time, data reduction analysis trainees read and study the data reduction protocol, meet with a manager or training specialist to highlight and clarify key points, step through examples of different types of events, and review pre-coded events on their own (asking questions when necessary). They then take two proficiency tests; each includes ten events, and feedback is provided after each. If scores on either proficiency test are satisfactory (i.e., typically 90%) and no systematic errors were observed, then the analyst can proceed with data reduction. If not, that analyst is removed from the project. Intra-rater agreement on classifying events was periodically assessed in the SHRP 2 study; as compared to an expert rater, the overall agreement was 88% for crash and near-crash events [51]. If there was more than one sequence in the crash event (e.g., a subject almost rear ends a lead vehicle and then is rear ended by the following vehicle), then the first sequence is defined as a near-crash and the second sequence is defined as a crash. A fixed number of short video baseline segments which include no crash-related events will be selected at random for each driver proportional to their contribution to the total distance travelled by the cohort. Data reduction will occur on these baseline segments in exactly the same manner as it occurs for the safety critical events.

#### Lane-keeping

The ability to maintain the vehicle’s movement within the appropriate lane, without deviating into other lanes or off the road, is a fundamental aspect of driving. We have selected lane-keeping as a focus because our previous driving performance studies indicate that lane-position is one of the most common problem areas for visually impaired drivers [54–57].

##### Data reduction

Lane-keeping, the ability to maintain the vehicle’s position within the selected lane, without deviating into other lanes or off the side of the road, is measured for any portion of roadway where the lane tracking machine vision software produces data with a high degree of confidence (i.e., where there are sufficient pavement markings). Although not all roadways have sufficient lane markings on the pavement to permit the successful generation of lane metrics, typically every participant drives on enough well marked roadways to get a good sample of these lane-keeping behaviors. Lane position is continually measured via VTTI’s custom machine-vision software which processes the forward video output, so its temporal frequency is that of the video itself (15 Hz). What is measured is the position of the vehicle with respect to the center of the lane and the lane markers. This measure, combined with road and vehicle width information, can be used to measure lane-keeping behavior for each driver in any number of ways defined by researchers, including, for instance, the number of times per mile where the vehicle exceeds the lane boundary or the degree of vacillation around the center of the lane (e.g., number of times per mile where the vehicle’s center crosses the lane center), etc.

#### Turning at intersections

The most common type of older driver collision involves intersections and while turning [58, 59]. Controlling a vehicle through an intersection is a complex visually guided behavior, relying on visual sensory function, visually processing speed and gaze strategies. Turning left at an unprotected intersection is an accentuated problem for older drivers in the US since it crosses oncoming traffic, yet we will study both right and left turns since both are common maneuvers in the course of everyday driving, and inclusion of both will allow us to compare driver behaviors in these two situations.

##### Data reduction

We will identify one or more intersections where a large number of participants have traversed along the same pathway at least three times; there are several candidate intersections since all participants drive to and from the Clinical Research Unit and the study garage. VTTI has developed protocols for determining glance behaviors across various locations inside as well as outside the vehicle [60]. Trained data-reductionists review the relevant epoch of video on a frame-by-frame basis to determine the glance location for each frame of video. From those base data, we will develop other metrics including glance path diagrams, glance location probabilities, and time on/off the forward roadway. Gaze behavior will begin being coded at 30 s prior to turn initiation (defined by the first behavioral indication that the driver intends to make a turn (e.g., turn signal initiation or entering a dedicated turn lane), then through the intersection’s “conflict zone” (i.e., the area where there could be a potential conflict between the subject vehicle and any other) and through turn conclusion (i.e., where the vehicle kinematics return to steady state conditions). We will examine the extent of head rotation of drivers as they move through intersections; this has previously been reported to differ between middle-aged and older drivers [20] and also differs for drivers among drivers with visual field loss [61]. VTTI has developed machine-vision software “the Mask” that can be used to measure head position and degree of rotation on a frame-by-frame basis when applied post-hoc to driver video. The application of this software leads to estimates of several lateral and longitudinal head rotation variables each of which can be duplicated in the longitudinal dimension (i.e., where the head is nodding up or down). The DAS measures speed via both GPS and from the vehicle’s network, allowing us to estimate intersection traversal time or speed.

#### Driving while engaging in secondary tasks

Performance of secondary tasks while driving (e.g., talking on a cell phone or texting) hamper driver safety and performance [23, 62]. Drivers using cell phones tend to take longer to react to relevant targets or events while driving and to recover their speed after braking, increase their following distance, reduce their speed, and miss traffic signals [63, 64]. Little is known about the impact of secondary tasks on older drivers, although recent studies have suggested that distractions from second task engagement by older drivers elevates crash risk and impairs driver performance in a driver simulator [23, 64–66]. This is not surprising since older adults tend to perform worse than younger adults in dual-task situations [67]; these deleterious effects may be accentuated for visually impaired older drivers. Besides cell phone use there are other types of secondary tasks drivers engage in – eating, smoking, adjusting controls on the radio, and interacting with pets in the vehicle.

##### Data reduction

Each crash-related event will be coded with respect to whether or not the driver’s engagement in a secondary task or other distraction was present during the event. VTTI has established standard protocols for coding different categories of secondary or distracting tasks while driving, with 67 distinct categories (e.g., talking on cell phone, texting, pet in vehicle, eating) [68].In order to determine whether secondary tasks elevate the risk of safety critical events, it is necessary to also have an estimate of the occurrence of such tasks during routine driving. It would be prohibitive to view all of the video-captured driving for each study participant; a more efficient technique for obtaining information on routine driving is to use sampling.

#### Driving with a “co-pilot”

There have been reports that older drivers tend to make use of co-pilots (person in the front passenger seat who alerts them to objects/events in the roadway and provide cues and information for navigation) [69, 70]. It remains to be determined to what extent co-pilots impact older driver safety and performance. Some studies have suggested that older adults have lower collision rates when passengers in the car [71–73]; but these studies do not address whether it is the co-piloting role of the front seat passenger that is protective. From our previous work on visually impaired drivers who use bioptic telescopes, some drivers report they value the presence of a normally sighted passenger in clarifying the roadway environment [55]; yet the drivers we studied were young-to-middle aged adults, not older adults. This study will be an opportunity to examine the impact of co-piloting on driving behavior in older drivers with vision impairment.

##### Data reduction

One of the video cameras is positioned to take a blurred still image of the cabin every ten minutes. The image is permanently blurred to protect the identity of unconsented passengers. This still image allows reductionists to determine the presence of other passengers, most importantly whether or not there is a “co-pilot” in the front passenger seat. While the mere presence of the passenger in the front seat does not necessarily mean that the passenger is actually performing “co-piloting” functions such as verbally pointing out obstacles, traffic control devices, potentially threatening roadway situations or assisting with navigation, we will be able to determine if the driver’s behavior and vehicle kinematics are different when a passenger is present versus when not. We will also attempt to make a determination of the passenger’s general age group and gender, as possible. We will compare trips which include a front passenger to those that do not in terms of glance and gaze-related behaviors, sudden acceleration/deceleration, and speed.

## Sample size estimation

Based upon prior work [54–56, 61], a common problem for visually impaired drivers is lane deviations and steering steadiness; therefore, it will be used to motivate the sample size calculations. Given a sample size of ~ 20 participants per category of participants (see Table 1), which sums to a total sample size of 195 participants, and an expected rate of lane deviations of 0.31/mile driven, we have approximately 70% power (α = 0.05, two-sided) to detect a 2-fold difference in lane deviations between drivers with and without vision impairment. For safety critical events, which have a lower incidence rate, the power to detect differences of a similar magnitude is less when using similar analytical approaches. However, the analysis of safety critical events is amenable to other study designs (e.g., case-crossover) that will yield increases in power and will be explored during the statistical analysis.

## Statistical analysis plan

The primary aim of the proposed work is to examine the relationships between vision and naturalistic driving performance in older drivers ≥70 years old. We will examine associations between different types of vision impairment and crash and near-crash involvement, lane-keeping, turning at intersections, driving performance under secondary task demands, and when a “co-pilot” is present. For these analyses, study participants will be grouped according to vision impairment status (e.g., whether impairment is present or not), type of impairment, impairment severity, and groups will be compared with respect to demographic, health and functional (including vision), behavioral and driving characteristics using a variety of statistical tests including analysis of variance and chi-square tests or their non-parametric equivalents and/or small-sample size (e.g., Freeman-Halton extension of Fisher’s exact test), as deemed necessary. The objective of these analyses is to identify potential confounders that might subsequently be used to adjust associations between the vision groups and the dependent measures of naturalistic driving.

For comparisons between the participant groups and the DAS-derived dependent variables,

it is important to keep in mind that the diversity in the measures will require a wide range of statistical approaches, the most appropriate of which may not be clear until the characteristics of the actual data have been evaluated and the analysis process is underway. It is important to keep in mind that the measurement of naturalistic driving data proposed is relatively novel, so there is little precedent to draw from. Simply based upon the nature of the measures described above, several approaches are likely to be appropriate and be employed. Because all of the dependent variables can be enumerated as counts (i.e., the # of times each event occurs), Poisson regression would be a useful tool to model the count of these events per mile driven as a function of vision impairment status with and without adjustment for potential confounding characteristics. Using this approach would call for the calculation of rate ratios and associated 95% confidence intervals using the unimpaired group as the common reference. Another, related, approach would be the use of generalized estimating equations to similarly model the occurrence of these events as a function of vision impairment status. This approach would model each event as a binary occurrence but account for the clustering of events within participants and/or drives. Odds ratios and associated 95% confidence intervals would be estimated for vision impairment as well as for other variables of interest.

The focus of our second aim is to explore the modifying effect of driver, environmental and vehicle factors on the association between vision impairment status and driving measures procured from the DAS. Therefore, to evaluate the presence of effect modification, the aforementioned statistical models will be stratified according to potential effect modifiers and the relevant measures of association (e.g., ORs) will be compared across strata.

Aim 3 seeks to examine the relationships between driving performance as measured by naturalistic driving methods and driving performance ratings provided by a CDRS on a standardized driving route (the clinical gold standard). This will provide the opportunity to examine the validity of CDRS ratings against objective measures of performance. Based upon prior work, many of the on-road driving performance ratings are ordinal variables, some of which may be used to classify drivers on a binary basis as “safe” or “unsafe”. However, in the context of the present study, the ordinal measures are likely of greater interest. As a result, we will calculate correlation coefficients (both Pearson’s and Spearman’s) for the association between the DAS- and CDRS-derived measures of driving performance which are common to both approaches, for example, lane-keeping. We will explore both the confounding and modifying influence of driver (including vision), environmental and vehicle factors on these associations using regression models using the DAS-derived measures as dependent variables and the CDRS-derived measures as independent variables. As noted above, the exact nature of these models will be highly reliant on the nature of the dependent variables; however, Poisson and logistic regression are two likely approaches.

## Discussion

Older drivers are the fastest growing group of drivers on the road in the US [74]. There are approximately 40 million adults aged ≥70 years old in the US (69) and 4 out of 5 of them (32 million), are drivers [74]. Older adults have a crash rate nearly equal to that of younger drivers whose crash rate is the highest among all age groups [75]. Once in a crash, older adults are more likely to be injured or die than are young drivers [76]. Removing the driver’s license of an older adult has negative consequences for the individual and the society (2–10). Identifying ways to enhance driver safety among older adults as well as identifying drivers who are unsafe behind the wheel has become a pressing public health issue, garnering much media attention.

Researchers focused on driver safety and performance have had access to several approaches:

epidemiological methods utilizing national crash databases, population-based surveys, statistical simulations, closed road circuits, laboratory-based studies on the characteristics of drivers, and driver simulator studies. The relative strengths and limitations of these research methods have been discussed at length previously [2]. These and other approaches have contributed substantially to the knowledge base. However, until recently there has been no feasible way to examine real-world, on-road driver behavior and vehicle kinematics in detail over extended periods of time. The naturalistic driving study paradigm has emerged to fill this gap facilitated by advances and miniaturization of computer, sensor, data storage, communications, and video technology. The measurement of actual, real-world driving behavior over extended periods of time, rather than short duration “snap-shots” of on-road driving, is the primary strength of the naturalistic driving approach. Limitations must also be acknowledged. For example, in our study generalization of findings from our older driver cohort to others remains unknown, an issue to be explored in future research. Volunteer bias is also present, however this is a problem for all older driver performance studies and is not unique to naturalistic driving studies. Some volunteers’ vehicles cannot be installed with a DAS because of incompatibilities between the designs of the DAS and vehicle. However, this rate is expected to be low, based on previous research.

In summary, the Alabama VIP Older Driver Study is the first naturalistic driving study whose design focuses on the examination of the association between vision impairment in older drivers and actual on-road driving, including both safety measures (crashes and near-crashes) and driver behaviors. Although a limited number of studies have examined the relationship between vision and driving in older adults using naturalistic techniques, the vast majority of drivers in previous studies had normal or near normal vision [15–21, 23], thus hindering an examination of the relationship. By design our study is not population-based but instead, focuses on those older drivers with vision impairment. Thus, the Alabama VIP Older Driver Study will provide novel information on how various types of vision impairments (e.g., contrast sensitivity loss, visual field impairment, slowed visual processing speed) and the severity of those impairments impact actual on-road performance and safety. Study findings have the potential to stimulate the development of improved methods for on-road evaluation of older drivers, rehabilitation interventions for visually impaired older drivers, and evidenced-based vision standard policies for licensure.

## Abbreviations

CDRS: Certified driving rehabilitation specialist
DAS: Data acquisition system
HFA: Humphrey Field Analyzer
Hz: Hertz
MVPT: Motor-free Visual Perception Test
SHRP 2: Strategic Highway Research Program 2
UAB: University of Alabama at Birmingham
VTTI: Virginia Tech Transportation Institute
